# Whole-exome sequencing reveals novel and previously reported variants in genes linked to white matter pathology in neurodevelopmental disorders

**DOI:** 10.1038/s41431-026-02182-y

**Published:** 2026-07-09

**Authors:** Naeem Ahmad, Tooba Aleem, Chunyu Liu, Maliha Rashid, Javeria Tehreem, Mary Ann Minal Junaid, Muhammad Farrukh Asif, Shakir Ullah, Sami Ullah, Tehseen Ullah Khan Afridi, Usman Raza Baig, Muhammad Tariq, Tahir Naeem Khan, Lydia Green, James A. Poulter, Naveed Altaf Malik

**Affiliations:** 1 National Institute for Biotechnology & Genetic Engineering College Pakistan Institute of Engineering & Applied Sciences (NIBGE-C, PIEAS) Jhang Road, Faisalabad, Pakistan; 2 Division of Molecular Medicine, Leeds Institute of Medical Research, University of Leeds, Leeds, UK; 3 International Peace Maternity and Child Health Hospital, Shanghai Key Laboratory of Embryo Original Diseases, School of Medicine, Shanghai Jiao Tong University, Shanghai, China; 4 Department of Molecular Biology and Genetics, National University of Medical Sciences, Rawalpindi, Pakistan; 5 Department of Paediatric Neurology, Leeds Teaching Hospitals Trust, Leeds, UK

**Keywords:** Genetics research, Genetics of the nervous system

## Abstract

Functional neuronal networks are essential for brain growth and development, with myelin or “white matter” playing a key role not just in neuronal transmission but also in axonal support, maintenance, and repair. Disruption of these networks through genetic variation contributes to a range of neurodevelopmental disorders, including both primary inherited white matter disorders and an ever increasing list of secondary leukodystrophies or leucoencephalopathies. Traditionally identified using neuroimaging techniques such as magnetic resonance imaging (MRI), clinico-radiological heterogeneity means MRI alone is rarely sufficient in providing a diagnosis requiring integration with genomic techniques and expert medical review. In this study, we describe an exome-first approach in a cohort of 78 consanguineous families with undiagnosed neurodevelopmental disorders in a healthcare resource-poor setting. The aim is to demonstrate that, even in the absence of MRI imaging, we have the potential to improve diagnostic accuracy and yield. We report positive findings in six families, including known variants in *MLC1, AIMP1, ASPA, and DEGS1*, and novel variants in *XPA* and *POLR3B*. This study highlights the effectiveness of whole-exome sequencing as a diagnostic tool for heterogeneous neurological disorders and provides additional evidence supporting gene-disease associations in conditions linked to white matter pathology.

## Introduction

Effective communication across brain regions is essential for normal brain function. Most of these communication pathways are provided by myelinated axons called the white matter that constitutes more than half of the brain’s structure. Abnormalities in white matter are associated with a broad spectrum of neurodevelopmental and neurodegenerative conditions [1]. These abnormalities have variable onset of disease symptoms and severity, but often result in significant morbidity and mortality in both children and adults [2]. Clinically, white matter disorders manifest with a variety of neurological symptoms, including developmental delay, spasticity, dystonia, nystagmus, behavioral change, and neuropsychiatric abnormalities [2–5].

Although MRI can identify abnormal T2 signal changes in the white matter, as well as other features such as cavitation and calcification to facilitate diagnosis of white matter pathologies and classification as hypomyelinating, dysmyelinating, or cavitating LDs, it is often insufficient to establish a specific molecular diagnosis [6]. Advances in sequencing technologies have transformed the diagnostic landscape, enabling the identification of genetic variants that underlie these conditions [7].

However, studies investigating the genetic basis of neurodevelopmental disorders have largely focused on well-characterized cohorts from high-resource settings. In contrast, few studies have explored the spectrum of genetic variants underlying neurodevelopmental disorders in Pakistan. The high rate of consanguineous marriage in this population increases the likelihood of identifying autosomal recessive pathogenic variants inherited from common ancestors [8]. In Pakistan, although specialized neurological care and advanced diagnostic facilities, including neuroimaging, are available, access to these services is often limited in rural populations due to geographic and socioeconomic factors. As a result, many affected individuals remain without a precise clinical or molecular diagnosis. This presents both a challenge and an opportunity for genetic studies aimed at improving disease characterization and variant interpretation, particularly in rare and ultra-rare disorders. As well as a diagnosis, the identification of a pathogenic variant informs future family planning following wider carrier testing.

In this study, we applied whole-exome sequencing (WES) as part of a larger investigation of 78 consanguineous families with undiagnosed neurodevelopmental disorders. From this cohort, we report six families in whom variants were identified in genes previously associated with abnormalities of the white matter.

## Materials and methods

### Study cohort and case selection

Families included in this study were derived from a larger cohort of 78 consanguineous families with predominantly undiagnosed neurodevelopmental disorders. These families were ascertained through field-based recruitment in different regions of Pakistan. Cases were selected based on the identification of rare variants in genes previously associated with white matter pathology, and inclusion was therefore driven primarily by genetic findings. Brain imaging was subsequently performed, where feasible, to support the genetic diagnosis.

### Sample collection and extraction of DNA

Peripheral blood samples were collected from affected individuals, their parents and normal siblings (where available) into sterile EDTA BD vacutainer^®^ tubes (Becton, Dickinson and Company, Franklin Lakes, NJ, USA). Genomic DNA was extracted using the GeneJET^TM^ Whole Blood Genomic DNA Purification Mini Kit (Thermo Scientific, K0782) following the manufacturer’s protocol. The quality and the quantity of the purified DNA were assessed using NanoDrop One (Thermo Fisher Scientific). DNA was stored at -20°C until further analysis.

### Genomic analysis

Whole-exome sequencing (WES) was performed on all affected individuals available from each family using the Twist Human Core Exome kit (Twist Biosciences) and Illumina Novaseq 6000 platform, generating 150 bp paired-end reads with 68–84× mean depth. Sequencing reads underwent quality control with FastQC, adapter and quality trimming with fastp, and alignment to the GRCh38 reference genome using BWA-MEM. Variant calling was carried out using the HaplotypeCaller function of GATK4 [9], and annotation with wANNOVAR and/or variant effect predictor. Exonic and splice-site variants with a minor allele frequency (MAF) < 0.01 were prioritized based on inheritance patterns (as determined by pedigree analysis) and known associations with neurodevelopmental disorders (NDDs). The list of variants retained after initial filtering is provided in Supplementary Table 1. To further prioritise variants in genes known to be associated with white matter disorders, we further filtered for genes present in the “Childhood onset leukodystrophy–V30.1 (R190)” panel in PanelApp (https://nhsgms-panelapp.genomicsengland.co.uk/panels/496/v30.1). Candidate variants were validated by Sanger sequencing, and segregation analysis was performed in all available family members. Variants were classified in accordance with ACMG guidelines using the Franklin interpretation platform (https://franklin.genoox.com—Franklin by Genoox), accessed on 12 April 2026. The detailed methodology is described elsewhere [10, 11].

## Results

### Family 1

**Fig. 1:**
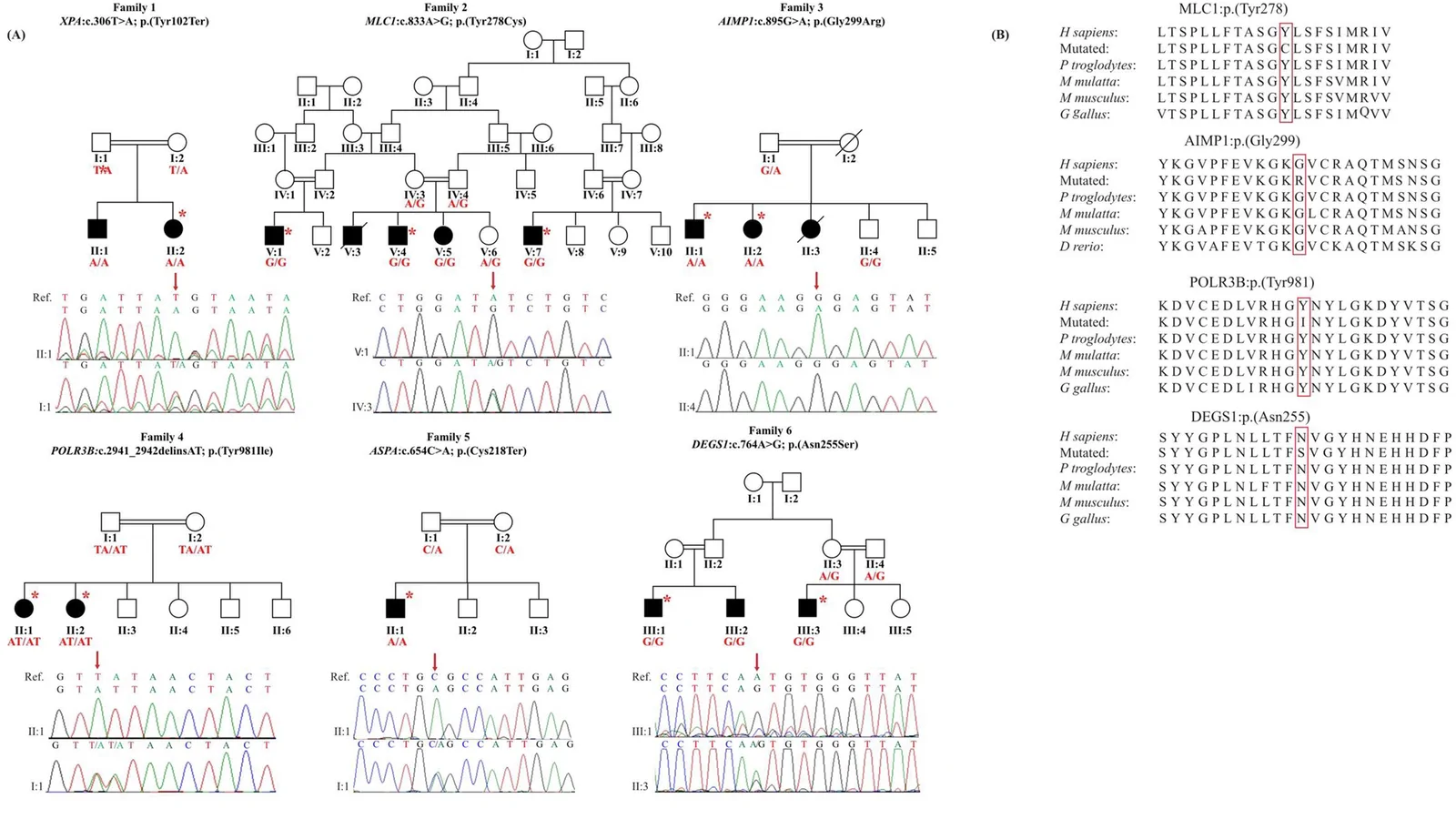
Pedigree, genetic findings and DNA Chromatograms in families with white matter disorders. **A** Upper panel: Pedigrees and genetic findings. For each family top bold text shows the family number and identified variant. Double horizontal lines in pedigrees indicate consanguinity; squares and circles symbolize males and females, respectively. Filled symbols indicate affected individuals; unfilled symbols indicate healthy individuals; diagonal line, deceased. Red asterisk indicates the individuals subjected to WES. The genotypes (red) for identified variants are indicated below each symbol where DNA was available. Lower panel: Sanger sequencing chromatograms of affected (upper) and healthy (lower) individuals with their IDs highlight the precise location (red arrow) of variants in the DNA sequence, reference sequence (Ref.) is written on top of chromatograms. **B** MLC1, AIMP1, POLR3B, and DEGS1 amino acid substitution variants lie in conserved regions of the respective proteins.

In Family 1, an 11-year-old male (II:1) and an 8-year-old female (II:2), born to consanguineous parents, presented with photosensitivity, developmental delay, and intellectual impairment (Fig. 1A). Both followed a similar disease trajectory, with the first cutaneous symptoms observed at around two months of age, progressing to recurrent severe sunburn of exposed skin over time. Other dermatological features included hyperpigmented macules and freckles. Developmental delay was global and significant, requiring full support for self-care. At the age of 11 years, the male sibling (II:1) had acquired a few indistinct words, whereas his sister (II:2) remained non-verbal at the age of 8 years. Although both achieved independent ambulation, this remained challenging due to the abnormal gait.

**Fig. 2:**
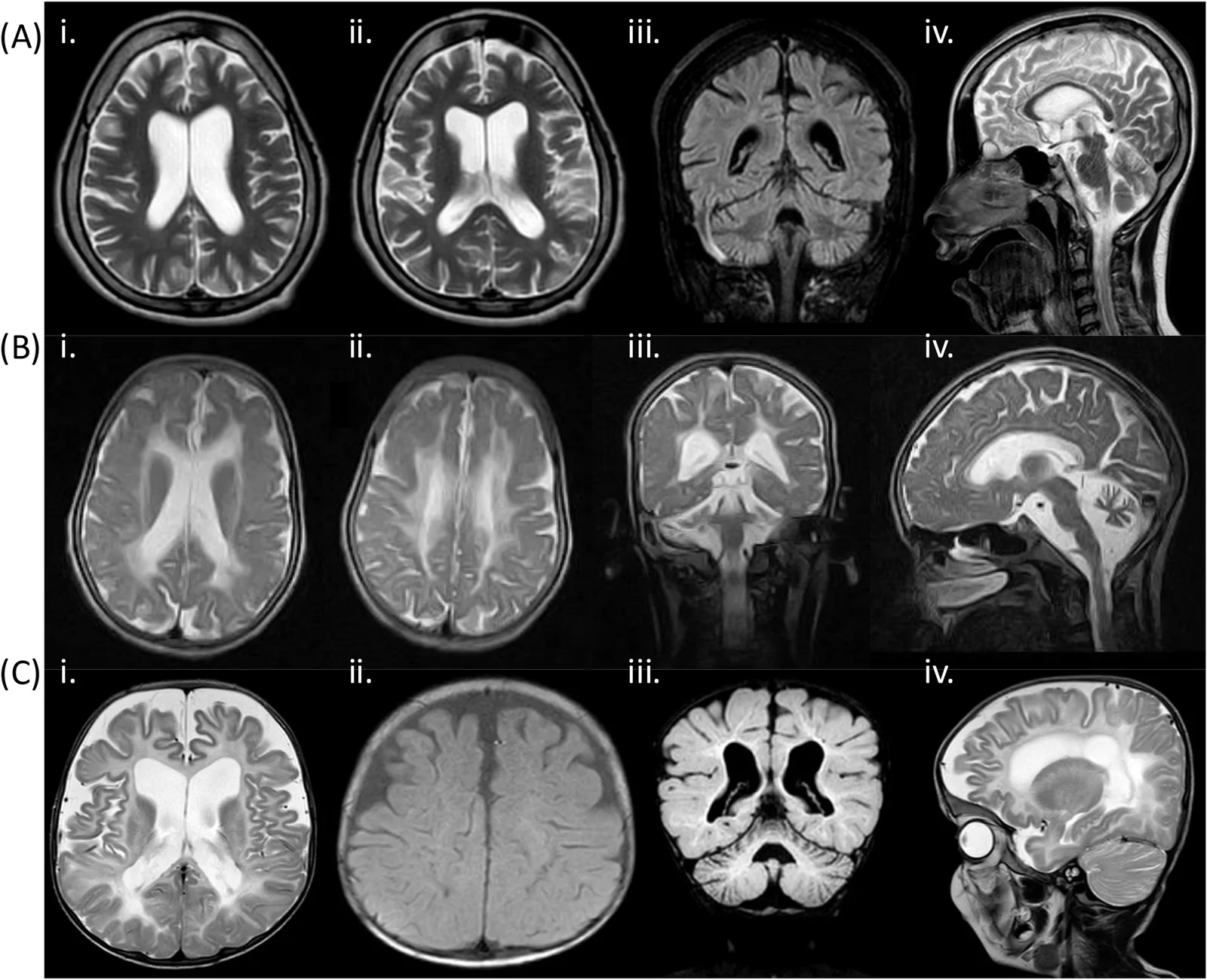
Brain MRI findings in affected individuals from different families. **A** *XPA* patient: (i, ii) Axial T2-weighted images demonstrating ventriculomegaly with prominent cortical sulci, consistent with cerebral atrophy. (iii) Coronal FLAIR image showing symmetrical prominence of cortical sulci. (iv) Sagittal T2-weighted image illustrating global cerebral atrophy with relative thinning of the corpus callosum. **B** *POLR3B* patient: (i, ii) Axial T2-weighted images demonstrating bilateral symmetrical hyperintensities in the periventricular white matter and centrum semiovale. (iii) Coronal T2-weighted image showing symmetric white matter involvement with ventriculomegaly. (iv) Sagittal T2-weighted image illustrating cerebellar atrophy with prominent folia and relative thinning of the corpus callosum. **C** *ASPA* patient: (i, ii) Axial T2-weighted images demonstrating diffuse, bilateral, and confluent hyperintensities involving the periventricular and deep white matter. (iii) Coronal FLAIR image showing symmetrical white matter involvement with ventriculomegaly. (iv) Sagittal T2-weighted image illustrating global involvement with an abnormal myelination pattern.

Magnetic resonance imaging (MRI) of the brain without contrast revealed no evidence of acute infarction, intracranial hemorrhage, or significant midline shift. Prominent sulci and dilated extra-axial cerebrospinal fluid (CSF) spaces were noted, consistent with cortical atrophy. Mild dilatation of the ventricular system, proportional to the degree of sulcal atrophy, was observed, with asymmetrically more prominent frontal horns compared to the occipital horns. The third and fourth ventricles were mildly enlarged along the midline. Prominent cerebellar folia and interfoliate sulci suggested mild cerebellar atrophy. The brainstem and proximal cervical cord were grossly unremarkable, with no evidence of tonsillar herniation. The corpus callosum exhibited a slight thinning at the distal body. Paranasal sinuses, mastoid air cells, and orbits were normal, with no destructive osseous lesions or significant abnormalities detected in the sella or pineal regions, indicative of cerebral and cerebellar atrophy. While some evidence of white matter volume loss was observed, no definite leukodystrophy-associated abnormalities were observed (Fig. 2A).

Exome sequencing identified a novel homozygous nonsense variant in *XPA*, ENST00000375128.5:c.306 T > A;p.(Tyr102Ter). The variant was confirmed by Sanger sequencing and segregated with the disease in all available family members (Fig. 1A&B). This premature stop-codon is predicted to be deleterious by MutationTaster, DANN and BayesDel; and has a CADD-Phred score of 35 (Supplementary Table 2). The variant is predicted to trigger nonsense-mediated mRNA decay (NMD), with an NMD-trigger probability of 0.758 (predNMD; https://compbio.berkeley.edu/proj/prednmd/). The variant (rs199710643) is rare, with a gnomAD v.4.1.0 (exomes) minor allele frequency of 0.000001371 (0.00002200 in the South Asian population) and was not listed in ClinVar. Notably, no homozygotes have been reported for this variant. A comprehensive review of the published literature and searches of disease-specific databases, including DisGeNet and LOVD version 3.0, was conducted. No publications or associations were found linking this *XPA* variant and disease. The variant was classified as pathogenic according to ACMG criteria (Table 1). A diagnosis for xeroderma pigmentosum complementation group A (OMIM #278700) was confirmed based on the genetic finding and observed clinical phenotype.

**Table 1:** List of likely disease-causing variants identified by WES in this study.

Family	Gene			ACMG Classification		CADD-Phred score	Reference
		Variant	Predicted impact	ACMG class	ACMG criteria		
1	*XPA* (NM_000380.4)	c.306 T > A; (p.(Tyr102Ter)	Stop gain	Pathogenic	PM3, PVS1, PM2	35	This study
2	*MLC1* (NM_015166.3)	c.833 A > G; (p.(Tyr278Cys)	Missense	Likely Pathogenic	PM3, PM2, PM1, PP2, PP3	23.7	This study, ClinVar (VCV000626279.4)
3	*AIMP1* (NM_001142416.2)	c.895 G > A; (p.(Gly299Arg)	Missense	Likely Pathogenic	PM3, PM2	32	(Iqbal et al., 2016)
4	*POLR3B* (NM_018082.6)	c.2941_2942delinsAT; p.(Tyr981Ile)	Missense	VUS	PM2, PP2	25.9	This study
5	*ASPA* (NM_000049.4)	c.654 C > A; p.(Cys218Ter)	Stop gain	Pathogenic	PM3, PVS1, PM2	36	This study, ClinVar (VCV000002608.23)
6	*DEGS1* (NM_003676.4)	c.764 A > G; (p.(Asn255Ser)	Missense	Likely Pathogenic	PM3, PM2	24.1	This study, ClinVar (VCV000625852.34)

### Family 2

Family 2 comprises an extended pedigree with a history of consanguineous marriages (Fig. 1A). The proband (V:1) is a 9-year-old male who presented with a prominent forehead, muscle contractures and joint stiffness predominantly in the lower limbs. His head circumference examination was 58 cm (+4.0 SD). He had an uneventful birth with normal early motor and cognitive development. A thorough investigation into his medical history revealed generalized tonic-clonic seizures at 6 years of age, after which he began to experience gait difficulties. He continued to follow a progressive course and was non-ambulant and dependent for all care at the time of assessment (aged 8 years). Brain MRI suggested white matter abnormalities: diffuse bilateral symmetric T2-weighted hyperintensity and T1-weighted hypointensity in central white matter, giving a swollen appearance to cerebellar white matter. Bilateral subcortical CSF- intensity cysts in the anterior temporal lobes and high frontal lobes of variable sizes were also noted alongside megalencephaly. Other affected individuals of the family had similar phenotypic features. The head circumference for V:4, V:5 and V:7 were +3.1, +6.0 and +3.0 SD, respectively. However, these individuals were unavailable for detailed clinical and neuroimaging assessments.

Exome sequencing of the proband and two affected siblings identified a homozygous missense variant in *MLC1*, ENST00000311597.10:c.833 A > G; p.(Tyr278Cys), which segregated in the family (Fig. 1A, B). This variant is listed in ClinVar (VCV000626279.4) as likely pathogenic and has a gnomAD v.4.1.0 (exomes) MAF of 0.000001368 (0.00002196 in the South Asian population). This variant has a CADD-Phred score of 23.7 and is predicted to be damaging by MutationTaster, DANN and Varity (Supplementary Table 2). Conservation analysis indicated that the variant lies in a conserved region of the protein adjacent to a transmembrane domain (Fig. 1C). This variant was classified as likely pathogenic according to ACMG criteria (Table 1). The genetic finding confirmed a diagnosis of megalencephalic leukoencephalopathy with subcortical cysts 1 (OMIM# 604004) in this family.

### Family 3

Family 3 presented with 31-year-old male (II:1) and 42-year-old female (II:2) siblings with severe intellectual disability (ID), aggressive behavior and neurological involvement (Fig. 1A). The male patient had more severe ID and aggression, he was unable to maintain personal hygiene or feed himself. His head circumference was 52 cm (-2.1 SD) at examination. He developed gait difficulties around 6 years of age, accompanied by progressive muscle spasticity more pronounced in the lower limbs, leading to complete loss of ambulation. Other neurological features included lack of speech and a history of epileptic seizures. The affected female had similar features, but the progression of the disease symptoms was considerably slower compared to her brother. She had severe ID, mild aggression, and developed gait problem at the age of 15 years. Now at the age of 42, she can still walk with an abnormal gait without support. She also had a history of epileptic seizures. Her head circumference at the time of examination was 52 cm (-2.2 SD). An elder female sibling, deceased at the age of 40 years, had the same phenotype and pattern of disease progression similar to her sister.

Exome sequencing of both affected individuals, looking for shared homozygous variants, identified a missense variant in *AIMP1*, ENST00000672341.1:c.895 G > A; p.(Gly299Arg). Sanger sequencing confirmed the variant segregated with the phenotype in available family members (Fig. 1A, B). This variant is not listed in ClinVar and has a gnomAD v.4.1.0 (exomes) allele frequency of 0.000004791 (0.00008787 in the South Asian population). This variant has a CADD-Phred score of 32, is predicted deleterious by MutationTaster, DANN, PolyPhen2, SIFT and Varity (Supplementary Table 2) and is located in a conserved region at the c-terminus of the protein (Fig. 1C). The variant was classified as likely pathogenic according to ACMG criteria (Table 1). A diagnosis for hypomyelinating leukodystrophy-3 (OMIM#260600) was made based on these genetic findings and the clinical symptoms observed in the patients.

### Family 4

In Family 4, two affected sisters, aged 10 and 8 years (II:1 & II:2), presented with similar clinical features (Fig. 1A). They were born to consanguineous parents and exhibited delayed developmental milestones and intellectual disability. Neither achieved independent ambulation or speech. They demonstrated generalised muscle stiffness and degeneration, predominantly in the lower limbs. Additional clinical features included strabismus, congenital cataracts, a protruding tongue and continuous drooling. Brain MRI of one individual (II:1) demonstrated cerebral and cerebellar atrophy with dilatation of the ventricular system. In addition, bilateral symmetrical T2-weighted hyperintensities were observed in the periventricular white matter and centrum semiovale (Fig. 2B).

Exome sequencing of both affected individuals revealed a novel two-base-pair substitution in *POLR3B*, ENST00000228347.9:c.2941_2942delinsAT;p.(Tyr981Ile). Sanger sequencing confirmed both affected siblings were homozygous and the parents were heterozygous for the variant (Fig. 1A, B). The variant was not listed on ClinVar nor in gnomAD v4.1.0, is located in a conserved region of the protein (Fig. 1C) and is predicted damaging by MutationTaster, PolyPhen2 and MutPred2. It has a CADD-Phred score of 25.9 (Supplementary Table 2) and was classified pathogenic by ACMG criteria (Table 1). This genetic finding and the observed clinical features informed a diagnosis for hypomyelinating leukodystrophy-8, HLD8 (OMIM# 614381) in this family.

### Family 5

In Family 5, a two-year-old male child (II:3) presented with an undiagnosed neurological disorder (Fig. 1A). At the time of assessment, he was unable to hold his head upright, sit, stand, or walk independently. His head circumference was 50.5 cm (91st percentile). Furthermore, he had a history of epileptic seizures starting at eight months of age, and typically lasted between 5 and 10 minutes. The seizures were resistant to common antiepileptic medications, including divalproex sodium and carbamazepine. Brain MRI demonstrated an abnormal myelination pattern not corresponding to age, with features suggestive of hypomyelination, along with cerebral and cerebellar atrophy characterized by prominent cortical sulci, dilated cerebellar folia, and proportionate ventriculomegaly (Fig. 2C).

Exome sequencing of the proband identified a known homozygous nonsense variant in *ASPA*, ENST00000263080.3:c.654 C > A;p.(Cys218Ter) which was confirmed by Sanger sequencing. Both parents were heterozygous for the variant (Fig. 1B). The variant is predicted to be deleterious by MutationTaster, DANN and BayesDel, with a CADD-Phred score of 36 (Supplementary Table 2), and likely to trigger NMD with a probability of 0.531 (predNMD; https://compbio.berkeley.edu/proj/prednmd/). According to ACMG criteria it was classified as pathogenic (Table 1). On the bases of these genetic findings and the clinical presentation of the patient, a diagnosis of Canavan disease (OMIM# 271900) was made.

### Family 6

In Family 6, three affected male individuals (III-1, III:2, and III:3, Fig. 1A) presented with a remarkably consistent and severe clinical phenotype, characterized primarily by profound congenital motor impairment without significant cognitive involvement. They exhibit a severe motor deficit, evident as a complete failure to achieve major motor milestones; none of the patients developed independent sitting, or the ability to walk. This motor deficit was compounded by the development of global muscle contractures and clinical signs of muscle degeneration, suggesting a progressive underlying process. Functional status was severely impacted, with two individuals (III-1, III:3) being bedridden and the third (III:2) retaining only the ability to crawl.

Beyond the gross motor deficits, the phenotype included slurred speech (dysarthria) in all three subjects, though without reported drooling. Furthermore, the patients were devoid of dysmorphic facial features, strabismus, or a history of seizures. Consequently, all individuals were fully dependent for activities of daily living but maintained the ability to feed orally. WES from two individuals from different loops (III:1, III:3) identified a previously reported (ClinVar:VCV000625852.34) pathogenic homozygous missense variant in *DEGS1* ENST00000323699.9:c.764 A > G;p.(Asn255Ser). The variant was confirmed and its segregation validated by Sanger sequencing all the available DNA samples from the family (Fig. 1A). This variant is present in conserved amino acid region of the protein (Fig. 1B) and in silico prediction tools including SIFT, PolyPhen2 and DANN classified this variant as disease-causing (Supplementary Table 2). This variant was classified as likely pathogenic according to ACMG criteria (Table 1). Based on the genetic finding and the phenotypic features, a diagnosis for hypomyelinating leukodystrophy 18 (OMIM#618404) was inferred.

## Discussion

White matter abnormalities are a common feature of a wide spectrum of neurodevelopmental and neurogenetic disorders, ranging from classical inherited white matter disorders (IWMDs), also known as leukodystrophies, to more heterogeneous conditions with variable white matter involvement. More than 100 different primary IWMDs exist, with pathogenic variants in approximately 270 genes, a number that increases substantially to over 2500 genes when any gene associated with changes in white matter is considered. These include genes associated with mitochondrial disorders, inborn errors of metabolism, cerebral calcification and intellectual disability. This extensive genetic and phenotypic heterogeneity presents a significant diagnostic challenge.

While brain MRI has improved the recognition and classification of white matter abnormalities, it is often insufficient to establish a definitive molecular diagnosis. The integration of genomic approaches, particularly whole-exome sequencing, has therefore become essential for identifying underlying genetic causes. These challenges are further compounded in populations with high rates of consanguinity and variable access to specialized healthcare, where many patients remain without a precise diagnosis. In this study, we applied a genotype-first approach and identified pathogenic variants in six families with neurodevelopmental disorders in genes linked to white matter changes, enabling a molecular diagnosis in all families.

In Family 1, we identified a novel truncating variant in *XPA*, which led to a diagnosis of xeroderma pigmentosum complementation group A (XP-A). XP is a rare autosomal recessive disorder marked by extreme sensitivity to ultraviolet (UV) light, significantly increasing the risk of skin cancers and neurological complications [12–14]. The identified variant introduces a premature stop codon, with the resulting mRNA likely to be either subjected to nonsense-mediated decay (NMD) or produce a truncated XPA protein with only 102 out of 273 amino acids. This truncation results in the loss of the major part of the protein, including the DNA binding domain (DBD), critical for nucleotide excision repair (NER) [15, 16].

The family’s clinical presentation aligns with known XP-A phenotypes, where approximately 25% of patients present with neurological involvement [17–19]. However, in this family, the neurological symptoms, including cognitive decline and motor dysfunction, appear to be relatively severe compared to previously reported cases. Although neurological involvement in XP-A has been associated with cerebral atrophy and, in some cases, white matter abnormalities, neuroimaging findings can be variable [17, 20]. In our case, brain MRI demonstrated cerebral and cerebellar atrophy, including white matter volume, but no overt T2 signal change. This pattern is in keeping with previously reported neuroimaging findings in XPA, where progressive cerebral atrophy predominates over leucoencephalopathy.

XP is classified into eight complementation groups (XP-A to XP-G) and a variant group (XP-V), each associated with mutations in different genes involved in NER. Of these, mutations in *XPA* are known to produce the most severe form of the disease with both skin and neurological complications. XPA plays a crucial role in the NER pathway, required for UV-induced DNA damage repair in sun exposed areas [12, 13]. Most of these roles require the DBD domain of XPA therefore the absence the DBD will lead to impaired NER and accumulation of UV-damaged DNA in sun exposed areas [21]. The diagnosis of XPA is therefore of particular importance in this case given the family resides in rural Pakistan, a region which experiences on average 8 hours of sunshine per day and has a UV index value > 5 (indicating high or very high UV exposure) for the majority of daylight hours.

In Family 2, we identified a known pathogenic missense variant in *MLC1*, resulting in a clinical diagnosis of megalencephalic leukoencephalopathy with subcortical cysts (MLC) type 1. The proband’s progressive neurological deterioration, megalencephaly, characteristic white matter abnormalities on MRI aligns with classic MLC1-related features [22], further supporting the pathogenicity of this variant. Furthermore, diffuse white matter abnormalities and subcortical cysts are prominent radiological features in MLC1-related MLC, consistent with the findings in this case [22, 23].

*MLC1* encodes a membrane protein predominantly expressed in astrocytes that plays a crucial role in maintaining the homeostasis of ions and water in the brain. Astrocyte vacuolation has been observed in cell and animal models of MLC, similar to the vacuolated white matter seen in patients with MLC1 [24]. However, this vacuolation is preceded by defects in the development of perivascular astrocytic processes, vascular smooth muscle cell maturation, CSF flux, and cerebral blood flow. Based on these observations, MLC is primarily regarded as a developmental disorder of the gliovascular unit [25]. Our study provides additional evidence supporting the involvement of *MLC1* variants in the Pakistani population.

In Family 3, a homozygous missense variant in *AIMP1* segregated with a severe neurological phenotype consistent with hypomyelinating leukodystrophy 3 (HLD3). While our patients exhibited neurological symptoms typical of HLD3 [26–28], confirmation of brain abnormalities such as hypomyelination and cerebral atrophy was not possible as the family did not wish to proceed with an MRI. However, given the well-established association of *AIMP1* variants with HLD3 and the consistent clinical presentation observed in the affected individuals, the genetic finding strongly supports the diagnosis.

*AIMP1* plays a critical role in maintaining neuronal integrity, and its disruption has been associated with neurodegeneration and motor dysfunction in both human studies and animal models [29]. Previous reports have demonstrated phenotypic variability in *AIMP1*-related disorders, including cases with milder presentations or atypical neuroimaging findings [27, 28, 30–35]. Notably, Iqbal et al. reported two unrelated families carrying the same *AIMP1* missense variant (p.Gly299Arg) presenting with severe intellectual disability and normal brain MRI, and suggested that this variant may result in partial loss of function [36].

In contrast, the individuals in our study carrying the same variant exhibited a more severe neurological phenotype, including microcephaly, progressive motor impairment, spasticity, and seizures. This variability in clinical presentation, despite the same underlying genetic variant, highlights the phenotypic heterogeneity associated with *AIMP1*-related disorders. Such differences may reflect the influence of additional genetic or environmental factors on disease expression.

In Family 4, a genetic diagnosis of hypomyelinating leukodystrophy 8 (HLD8) was made following the identification of a novel two-base-pair substitution in *POLR3B*, supported by the observed clinical features. The availability of neuroimaging data from one patient further supports the diagnosis, with MRI demonstrating cerebral and cerebellar atrophy along with bilateral symmetrical white matter signal changes, consistent with previously reported features of *POLR3B*-related hypomyelinating leukodystrophy. HLD8 is marked by hypomyelination, hypodontia and hypogonadotropic hypogonadism, collectively defining 4H syndrome [37, 38]. Both affected siblings presented with developmental delay, muscle contractures and lack of speech; despite hypodontia being frequently observed in HLD8, this phenotype was absent in both siblings. At the time of examination, the patients were 10 and 8 years old, respectively, and had not yet experienced menarche; however, given their young age, hypogonadism was not initially suspected. The diagnosis of HLD8 in this family is therefore of clinical importance to facilitate and direct ongoing clinical care of both affected siblings.

The pathophysiology of POLR3-related leukodystrophies is not completely understood; however, one of the primary mechanisms involves the disruption of tRNA synthesis. Mutations in *POLR3* genes lead to abnormal transcription of tRNA, which is critical for protein synthesis and cellular function, particularly in the central nervous system (CNS). The relative abundance of certain tRNAs in the CNS compared to other tissues may contribute to the specific neurological manifestations observed in patients with POLR3-related leukodystrophies [39].

In Family 5, a homozygous pathogenic stop-gain variant in ASPA, predicted to result in nonsense-mediated decay or premature termination of translation after 217 amino acids, was identified in a two-year-old male with developmental delay and severe epileptic seizures, consistent with a diagnosis of Canavan disease (CD). The ASPA enzyme catalyzes the hydrolysis of N-acetylaspartate (NAA), and its deficiency leads to NAA accumulation, spongy white matter degeneration, and progressive neurological deterioration characterized by macrocephaly, psychomotor delay, seizures, and severe cognitive impairment [40]. Macrocephaly, typically defined as a head circumference exceeding the 97th percentile, is a common feature of CD; however, our patient, with a head circumference of 50.5 cm (91st percentile) at two years of age, did not meet the criteria for macrocephaly. Other features, such as severe seizures, were consistent with CD, and follow-up evaluations will be necessary to monitor his cognitive development.

The clinical features observed in Family 6 are consistent with the diagnosis of hypomyelinating leukodystrophy 18 (HLD18) caused by biallelic *DEGS1* variants. The profound congenital motor impairment, progressive contractures, and absence of dysmorphism align well with previously described *DEGS1*-related presentations, where early motor arrest and severe neuromuscular dysfunction are defining features [41, 42]. The identification of the known pathogenic variant c.764 A > G; p.Asn255Ser, reported in association with HLD18 [43] and functionally shown to reduce DEGS1 enzymatic activity, further supports the diagnosis [42]. The MRI, performed after confirmation of the genetic diagnosis, demonstrated hypomyelination and cerebral atrophy, providing further radiological evidence to support the diagnosis.

The phenotypic spectrum of our cases and previously published individuals with the same *DEGS1* variant (p.Asn255Ser) exhibits both overlap and significant differences. Patients in the cohort described by Dolgin et al. [43] showed a progressive neurological presentation marked by somewhat normal early development followed by halt in development course, gait impairment, and white-matter abnormalities and delayed myelination on MRI. The three affected siblings in our family, on the other hand, showed a far more severe congenital and primarily motor phenotype, characterized by profound hypotonia, early development of global contractures, total failure to reach basic milestones like independent sitting or walking, and progressive muscle degeneration that left two of them bedridden. Seizures were also absent in our patients which are frequently associated with *DEGS1*-related leukodystrophy. These variations imply that although *DEGS1*-dependent sphingolipid metabolism is consistently disrupted by the Asn255Ser variant, the degree and timing of clinical involvement may differ among individuals, possibly due to changing genetic or environmental factors. No brain MRI data were available for this family to assess white matter involvement. Despite this limitation, our findings provide additional clinical observations associated with the DEGS1 p.(Asn255Ser) variant and further highlight the variability in phenotypic presentation.

A limitation of this study is the small cohort size, which limits broader conclusions regarding phenotypic variability. In addition, neuroimaging data were not available for all families, restricting genotype–phenotype correlation in some cases. Furthermore, the genotype-first design, with case selection based on genetic findings rather than uniform clinical criteria, may introduce variability in phenotypic characterization. Despite these limitations, our findings provide additional evidence supporting gene–disease associations in an underrepresented population.

In conclusion, we report two novel and four previously reported pathogenic variants in genes associated with neurodevelopmental disorders with white matter changes, enabling molecular diagnosis in all families. These findings have informed the clinical care of the affected individuals, in particular those in Family 1 and Family 4, and underscore the importance of genetic testing for the diagnosis of heterogeneous neurodevelopmental disorders. Our study highlights the importance of genomic approaches in settings where detailed clinical and radiological data may be limited.

## Supplementary information

Supplementary Table 1

Supplementary Table 2

## Data Availability

The variants reported in this study have been submitted to ClinVar (submission ID: SUB15047265).
