# Supplementary material for: Whole-exome sequencing reveals novel and previously reported variants in genes linked to white matter pathology in neurodevelopmental disorders
Source: Eur J Hum Genet. 2026 Jul 9;34(10):1465–72. doi: 10.1038/s41431-026-02182-y (PMC13634037; doi:10.1038/s41431-026-02182-y)
Supplement: Supplementary file 1 [file 41431_2026_2182_MOESM2_ESM.docx]

| Supplementary Table 2: *In silico* pathogenicity prediction scores of the variants identified by WES in this study. | | | | | | | | | |
| --- | --- | --- | --- | --- | --- | --- | --- | --- | --- |
| **Variant** | **Family ID** | **MutationTaster** | **DANN** | **MutPred2** | **BayesDel** | **PolyPhen2** | **SIFT** | **Varity** | **CADD-Phred Score** |
| *XPA*:c.306T>A; p.(Tyr102Ter) | Family 1 | Deleterious (1) | Deleterious (0.99) | NA | Deleterious (Strong) (0.65) | NA | NA | NA | 35 |
| *MLC1*:c.833A>G; p.(Tyr278Cys) | Family 2 | Deleterious (1) | Deleterious (1) | NA | NA | Deleterious (1) | Uncertain (0.001) | Deleterious (0.64) | 23.7 |
| *AIMP1*:c.895G>A; p.(Gly299Arg) | Family 3 | Deleterious (1) | Deleterious (1) | NA | NA | Deleterious (1) | Deleterious (0) | Deleterious (0.92) | 32 |
| *POLR3B*:c.2941_2942delinsAT; p.(Tyr981Ile) | Family 4 | Deleterious (1) | NA | Deleterious (0.93) | NA | Deleterious (0.815) | NA | NA | 25.9 |
| *ASPA*:c.654C>A; p.(Cys218Ter) | Family 5 | Deleterious (1) | Deleterious (0.99) | NA | Deleterious (Strong) (0.66) | NA | NA | NA | 36 |
| *DEGS1*:c.764A>G; (p.(Asn255Ser) | Family 6 | Deleterious (1) | Deleterious (1) | NA | Benign  (Supporting) (-0.28) | Possibly damaging (0.582) | Deleterious(0) | Deleterious  (low) (0.37) | 24.1 |
